# Sexual and Affectionate Behaviors and Satisfaction for Adults in Romantic Relationships: A Latent Profile Analysis

**DOI:** 10.1007/s10508-024-03016-y

**Published:** 2024-10-15

**Authors:** Alyssa N. Clark, Eva S. Lefkowitz

**Affiliations:** 1 Department of Psychology, The College of Wooster, 1189 Beall Ave., Wooster, OH 44691 USA; 2 Department of Human Development and Family Sciences, University of Connecticut, Storrs, CT USA

**Keywords:** Romantic relationships, Sexual behavior, Affectionate behavior, Latent profile analysis, Sexual orientation, LGBTQ+

## Abstract

Engaging in both sexual and affectionate behaviors with a romantic partner is often beneficial for adults’ sexual and relationship satisfaction and promotes relationship stability. However, prior research has primarily examined either adults’ sexual or affectionate behaviors, and has yet to explore patterns of sexual and affectionate behaviors and their associations with sexual and relationship satisfaction. In the current paper, we used a person-centered approach and latent profile analysis to identify specific profiles of adults’ physical behaviors in same-gender and mixed-gender relationships, and examined associations of these profiles with sexual and relationship satisfaction. Adults (*N* = 336, 85.4% LGBTQ+; 45% women, 30% men, 38.6% gender-diverse; *M*_*age*_ = 29.07 years) who were currently in a committed romantic partner relationship for at least six months completed online surveys. We found that a 3-profile solution best fit the data and identified the following profiles: Infrequent Physical Behaviors, Affection-focused Behaviors, and Comprehensive Physical Behaviors. Adults in the Infrequent Physical Behaviors profile were less sexually and relationally satisfied than adults in the other profiles. Adults in the Comprehensive Physical Behaviors profile were more sexually satisfied than the two other profiles. Further, profiles did not differ for mixed-gender compared to same-gender or gender-diverse couples. Our findings have implications for understanding the diversity in adults’ physical behavior patterns, including how clinicians might better support adults’ sexual and relationship satisfaction.

## Introduction

Engaging in sexual and affectionate physical behaviors in a romantic relationship is particularly beneficial for adults’ satisfaction, relationship stability, life satisfaction, and decreased bodily stress responses (Floyd, 2019; Gulledge et al., 2007; Lodge, 2015; Sprecher et al., 2018). Social exchange theory suggests that sexual and affectionate behaviors are often rewarding within relationships (e.g., Lawrance & Byers, 1995), but adults may not perceive all physical behaviors as equally sexually and relationally rewarding. Specific patterns of physical behaviors may also differ based on couple gender configuration (i.e., the combination of both partners’ gender identity, such as man/man, woman/gender-diverse partner, etc.). For instance, adults in same-gender or gender-diverse relationships might be more likely to have patterns of sexual behaviors that include less stereotypically heteronormative behaviors (i.e., behaviors other than penetrative penile-vaginal sex), as these adults may be anatomically unable, or uninterested in engaging in penetrative penile-vaginal sex (Diamond & Blair, 2018; Lindley et al., 2021).

For the purposes of this paper, because we have a primarily LGBTQ+ sample—individuals often underrepresented in prior similar research—we consider adults’ gender identity rather than sex assigned at birth in examining couple gender configuration. Sex assigned at birth may not align with an individual’s gender identity, and thus may not accurately represent their lived experiences (Diamond & Blair, 2018; Dyar et al., 2020). In contrast, determining couple gender configuration with gender identity might better reflect each partner’s lived experiences, especially when considering adults in same-gender or otherwise LGBTQ+ relationships.

Although much prior work on couples has considered either sexual behaviors or affectionate behaviors, it is important to examine a range of physical behaviors that include both sexual and affectionate behaviors together, as adults may find certain patterns of behaviors more sexually and relationally rewarding than others. For example, adults may be more satisfied if their physical behaviors include both frequent sexual and affectionate behaviors rather than only one or the other. Further understanding the associations of patterns of sexual and affectionate behaviors with satisfaction is important, as these associations may help clinicians better support their clients’ sexual pleasure and relationship enjoyment (Herbenick et al., 2010; Roels & Janssen, 2020). Of note, we consider any behavior that includes some aspect of physical touch with a romantic partner, whether sexual (e.g., genital touching) or affectionate (e.g., holding hands) a physical behavior, and focus on behaviors in the context of a romantic relationship. Thus, in the current paper we use a person-centered approach, which allows us to examine participants’ membership in patterns across multiple behaviors, rather than focusing on each behavior separately or as a mean across behaviors, to examine sexual and affectionate behaviors. Such an approach allows for a more nuanced understanding of physical behaviors. Specifically, we identify patterns of sexual and affectionate behaviors and consider how sexual and relationship satisfaction differ by patterns, and whether adults’ behaviors differ based on their couple gender configuration.

### Associations Between Physical Behaviors and Satisfaction

According to social exchange theory, partners maintain and form relationships through the process of relational exchanges (Kelley & Thibaut, 1978; Thibaut & Kelley, 1959). Adults in romantic relationships can perceive physical behaviors as rewarding or costly (Byers & Wang, 2004; Sprecher, 1998). Within romantic relationships, adults are motivated to engage in physical behaviors they find rewarding (e.g., indicate love, physical affection, or intimacy) while avoiding physical behaviors they deem costly (e.g., indicate conflict or a lack of intimacy; Lawrance & Byers, 1995; Levinger, 1976; Sprecher, 1998; Thibaut & Kelley, 1959). Engaging in physical behaviors also has a consistent and strong association with long-term relationship outcomes, such as sexual and relationship satisfaction (Sprecher et al., 2018). Despite this association, few researchers have investigated whether specific patterns of physical behaviors are associated with sexual and relationship satisfaction (see for exceptions, Frederick et al., 2017; Jawed-Wessel et al., 2019), which may reveal associations that variable-centered analyses cannot.

#### The Association Between Physical Behaviors and Sexual Satisfaction

Social exchange theory suggests that adults should perceive sexual and affectionate behaviors as sexually rewarding (Byers & Wang, 2004; Gulledge et al., 2007). Indeed, adults who engage in sexual behaviors—which we define as any physical behaviors intended to sexually arouse or fulfill sexual desire from the giver and/or in the recipient—more frequently feel more sexually satisfied in their romantic relationships than adults who engage in sexual behaviors less frequently (e.g., Blumenstock et al., 2020; Cohen & Byers, 2014; Heiman et al., 2011). Similarly, affectionate behaviors—which we define as any physical behaviors intended to show love, care, or affection from the giver and/or in the recipient—are also associated with sexual satisfaction. For instance, heterosexual adults who cuddle and are more affectionate after sex with a romantic partner are more sexually satisfied than adults who do not cuddle and are less affectionate after sex (Frederick et al., 2017; Muise et al., 2014). Heterosexual women who hug and kiss more during sex with a romantic partner are also more sexually satisfied than women who hug and kiss less during sex (Vannier et al., 2017). However, it is unclear whether sexual satisfaction differs by specific patterns of sexual and affectionate behaviors, especially when considering sexual and affectionate behaviors together.

Prior research on associations between specific sexual behaviors and sexual satisfaction has primarily examined only a few behaviors (i.e., oral sex, penile-vaginal sex, penile-anal sex; Blumenstock et al., 2020; Frederick et al., 2017). These results suggest that cisgender heterosexual couples who have oral or penile-vaginal sex more frequently are more sexually satisfied than their peers who have oral or penile-vaginal sex less frequently (Blumenstock et al., 2020; Frederick et al., 2017). Additionally, cisgender heterosexual women who engage in more frequent kissing, vaginal fingering, penile-anal sex, and partnered sex toy use are more sexually satisfied than women who engage in these behaviors less frequently (Blumenstock et al., 2020; Jawed-Wessel et al., 2019). Similarly, research on specific affectionate behaviors and sexual satisfaction has also only examined a few behaviors (i.e., cuddling, kissing, massaging; Herbenick et al., 2019). Cisgender heterosexual men and women who cuddle more frequently are more sexually satisfied than men and women who cuddle less frequently (Herbenick et al., 2019). However, it is less clear whether affectionate behaviors are only associated with sexual satisfaction when they occur in the context of sex, or if they are associated with sexual satisfaction more generally. Understanding whether sexual satisfaction differs by patterns of affectionate behaviors is important, as affectionate behaviors frequently occur independently from sexual behaviors (Gulledge et al., 2004; Jolink et al., 2021; van Anders et al., 2013). Therefore, affectionate behaviors might independently benefit adults’ sexual satisfaction, even when performed outside of sexual situations, perhaps by promoting relationship intimacy that may strengthen emotional bonds (Gulledge et al., 2007).

It is also unclear whether adults’ patterns of sexual and affectionate behaviors differ by couple gender configuration. For instance, woman/woman couples might engage in less frequent penetrative sexual behaviors (such as vaginal penetration with fingers, hands, or sex toys) but more frequent affectionate cuddling than man/man or man/woman couples (Diamond & Blair, 2018). Prior research has primarily examined cisgender heterosexual couples, however, and there is some evidence to suggest adults in same-gender or gender-diverse (e.g., women/non-binary partner) relationships might prefer sexual and/or affectionate behaviors that do not align with stereotypically heterosexual behaviors (Diamond & Blair, 2018; Lindley et al., 2021). For instance, engaging in penetrative behaviors may be unwanted or feel dysphoric for non-binary or transgender partners (Dyar et al., 2020). However, we know of little research that has examined whether patterns of sexual and affectionate behaviors vary by couple gender configuration, especially for gender-diverse couples, and whether sexual satisfaction differs by these patterns. Considering that couples commonly seek therapy or counseling for issues related to sexual behaviors and satisfaction, it is important to better understand what physical behavioral patterns might contribute to feeling less sexually or relationally satisfied (Blair & Pukall, 2014; Metz & McCarty, 2007; Whisman et al., 1997).

#### The Association Between Physical Behaviors and Relationship Satisfaction

Social exchange theory suggests that adults in romantic relationships would consider sexual and affectionate behaviors relationally rewarding (Byers & Wang, 2004; Gulledge et al., 2007; Sprecher, 1998). Indeed, adults who engage in sexual behaviors more frequently are more relationally satisfied than adults who engage in sexual behaviors less frequently (Lodge, 2015; Muise et al., 2016; Rausch & Rettenberger, 2021; Sprecher et al., 2018). Similarly, adults who engage in more frequent affectionate behaviors are more relationally satisfied than adults who engage less frequently in affectionate behaviors (Brashier & Hughes, 2012; Horan & Booth-Butterfield, 2010; Kent & El-Alayli, 2011; Schoenfeld et al., 2017).

However, less evidence indicates whether relationship satisfaction differs by specific patterns of sexual and affectionate behaviors. For example, there is some evidence that adults in heterosexual relationships who engage in more frequent penile-vaginal intercourse, oral sex, and manual stimulation are more relationally satisfied than adults who engage in these behaviors less frequently, but that frequent penile-anal intercourse is not associated with relationship satisfaction (Blumenstock et al., 2020). For affectionate behaviors, adults who hug and kiss more frequently are more satisfied with their relationship than adults who hug and kiss less frequently (Vannier et al., 2017; Wlodarski & Dunbar, 2013). Similarly, in an experimental study, mixed-gender couples who increased their frequency of kissing were more relationally satisfied than mixed-gender couples who did not (Floyd et al., 2009). However, to our knowledge, prior research has not examined whether additional sexual and affectionate behaviors are associated with adults’ relationship satisfaction, or whether relationship satisfaction differs by patterns of sexual and affectionate behaviors. In addition, it is important to consider different couple gender configurations. Thus, exploring whether adults’ sexual and affectionate behaviors differ by couple gender configuration and whether relationship satisfaction differs by patterns of sexual and affectionate behaviors may help clinicians better promote relationship satisfaction.

### Using a Person-Centered Approach to Studying Physical Behaviors

Although prior research has examined sexual and affectionate behaviors, many studies only examine sexual or affectionate behaviors and include few behaviors. These variable-centered approaches provide information about associations between individual behaviors and satisfaction, or average behaviors and satisfaction. Such variable-centered approaches rely on averages and have an underlying assumption that findings apply to all people (Bergman et al., 2003), and may not capture the complexity of how sexual and affectionate behaviors co-occur. In contrast, person-centered approaches can identify groups or classes of participants who display patterns of behaviors that are similar to each other but differ from the patterns of behavior of participants in other classes (von Eye & Bergman, 2003). These person-centered analyses, such as latent profile analysis (LPA), define the unit of analysis as the individual as a whole (Collins & Lanza, 2010), and examine response patterns at the individual level (Jung & Wickrama, 2008). Thus, person-centered approaches are a more holistic way to examine response patterns (Sianko & Kunkel, 2022).

Specific to our analyses, using a person-centered approach with a range of sexual and affectionate behaviors can present a more accurate and representative understanding of how sexual and relationship satisfaction differ by sexual and affectionate behaviors (e.g., McGuire & Barber, 2010). For example, it is possible that adults who frequently engage in many sexual and affectionate behaviors experience more sexual and relationship satisfaction than adults who engage less frequently in these behaviors, or in fewer behaviors overall. On the other hand, it is possible that in the presence of frequent sexual behaviors, the frequency of affectionate behaviors matters less for satisfaction. There may also be unique profiles of sexual and affectionate behaviors that involve sub-groups of behaviors beyond whether each category is overall frequent or infrequent, that are associated with better or worse satisfaction. A person-centered approach allows for a more nuanced understanding of physical behaviors. Variable-centered approaches group behaviors together, assuming behaviors are related to and contribute equally to satisfaction. In contrast, a person-centered approach can identify unique patterns of specific behaviors (Furr & Funder, 2004; Howard & Hoffman, 2018), and thus provide more detailed information about how sexual and relationship satisfaction differ by behavioral patterns.

### The Current Paper

In the current paper, we used a person-centered approach to examine latent profiles of sexual and affectionate behaviors for adults in different couple gender configurations. We also explored how sexual and relationship satisfaction differ by profiles of sexual and affectionate behaviors for adults in romantic relationships. Specifically, we had the following aims: 1) To use LPA (Lanza et al., 2013) to uncover profiles of sexual and affectionate behaviors for adults in romantic relationships. Due to the exploratory nature of LPA, we made no a priori predictions of the number of particular profiles. Yet, we expected profiles to be formed based on primarily high or low frequency of both sexual and affectionate behaviors. 2.) To examine whether latent profiles of adults’ sexual and affectionate behaviors differ by couple gender configuration. 3.) To examine how sexual and relationship satisfaction differ by latent profiles of sexual and affectionate behaviors for adults in romantic relationships.

## Method

### Participants and Procedure

We invited adults to complete an online survey about “adults’ romantic relationships and physical behavior.” Participants had to be in a current romantic relationship for at least six months, be fluent in English, and be at least 18 years old. We advertised the study through the authors’ accounts and/or contact information on several websites (e.g., Tumblr, Twitter, Reddit), and academic listservs, and recruited participants with both non-LGBTQ+ targeted and LGBTQ+ targeted survey descriptions. Overall, 382 participants completed the online Qualtrics survey in August and September 2022, which took about 25–35 min. Participants who completed the survey could enter a raffle for one of five $20 Amazon gift cards. We excluded participants who indicated they were currently in a long-distance relationship and were unable to report on physical behaviors with their partner in the last month (*n* = 3) or who did not respond to all physical behavior and satisfaction measures (*n* = 43). Thus, the final analytic sample consisted of 336 adults (*M*_age_ = 29.07 years, *SD* = 8.79, range = 18–71), the majority of whom lived in the United States (78%). Forty-five percent of the sample identified as women (30.4% as men, 38.6% gender-diverse). Participants’ relationship length ranged from 0.5 to 42.8 years (*M* = 6.4 years, *SD* = 6.1). See Table 1 for additional demographics.

**Table 1:** Sample demographics

		Total sample %
Transgender identity	Transgender	29.5%
Gender identity	Woman	45.5%
	Man	30.4%
	Non-binary	27.4%
	Genderqueer	13.1%
	Agender	6.0%
	Other	6.8%
Sexual orientation	Bisexual	33.4%
	Heterosexual/straight	14.6%
	Queer	11.9%
	Gay	11.9%
	Asexual/demisexual	11.0%
	Pansexual/omnisexual	8.7%
	Lesbian	6.3%
	Other	2.1%
Romantic orientation	Biromantic	34.5%
	Heteroromantic	21.0%
	Homoromantic	20.7%
	Panromantic/omniromantic	13.7%
	Aromantic/demiromantic	3.7%
	Other	6.4%
Ethnicity race	Hispanic/Latinx	7.9%
	White	91.5%
	Asian	6.2%
	Native American	2.0%
	Black	1.6%
	Middle Eastern	1.3%
	Other	3.0%
Relationship status	Dating	47.0%
	Married	32.1%
	Engaged	13.1%
	Other	7.7%
Partner transgender identity	Transgender	18.6%
Partner gender identity	Man	65.5%
	Woman	23.8%
	Non-binary	13.1%
	Genderqueer	6.3%
	Agender	2.1%
	Other	3.6%
More than one romantic partner(s)		10.1%

Due to missing data, *N* ranges from 325 to 336. Participants could select more than one gender and racial identity, and therefore these identities add up to more than 100%

### Measures

#### Demographics

Participants reported their age, race/ethnicity, gender identity, transgender identity, sexual orientation, romantic orientation, current relationship status, relationship length (in years and months), gender and transgender identity of their current romantic partner, and whether they had more than one partner. We directed participants to respond to subsequent questions based on their relationship with their primary partner or the partner they have been with the longest.

#### Couple Gender Configuration

We used the gender identity question to categorize participants as man, woman, or gender diverse. Of note, we did not differentiate individuals based on transgender identity (e.g., we grouped transgender men and cisgender men together). We then sorted participants and their partners into one of four couple-gender configuration groups: man/man, woman/woman, man/woman, and gender diverse (i.e., at least one partner identified as gender diverse). To include couple gender configuration in our final model, we created three dichotomous variables: *man/man* (1 = yes, 0 = no; 18.2%), *woman/woman* (1 = yes, 0 = no; 6.8%), and gender diverse (1 = yes, 0 = no; 30.1%), with the reference group adults in man/woman romantic relationships (44.9%).

#### Physical Behavior Inventory

To measure the frequency of sexual and affectionate behaviors, we used the Physical Behavior Inventory (PBI; Clark & Lefkowitz, 2024), a measure designed to assess sexual and affectionate behaviors for all individuals regardless of their sex assigned at birth or gender identity. Participants responded to the prompt: "In the last month, how frequently have you engaged in any of the following physical behaviors with your primary romantic partner?” on an 8-point Likert scale ranging from 0 = *Not at all* to 7 = *More than once a day.* Items that include behaviors with a vagina had an additional response option: “My partner and I do not have the genitalia to engage in this behavior.” We considered responses to any vaginal sexual behavior items of “My partner and I do not have the necessary genitalia” as missing data. To reduce participant bias, we presented 24 items for the PBI in randomized order. We presented the two items related to vaginal behaviors last because they had an additional scale point. The PBI has two behavior subscales. In the current paper, we wanted all participants regardless of gender identity or couple gender configuration to be able to engage in any behavior regardless of their anatomy. Thus, we computed penetration with hands/fingers, and penetration with penis, sex toy, or strap-on, by using participants’ response for whichever behavior (anal or vaginal) they more frequently reported. The final measure included an 11 item sexual behavior subscale (α = 0.92) and the 13 item affectionate behavior subscale (α = 0.90; see Table 2 for list of all behaviors).

**Table 2:** Item response means for latent profiles of physical behaviors

	Profile 1 Infrequent physical behaviors (15.96%)	Profile 2 Affection-focused behaviors (35.84%)	Profile 3 Comprehensive physical behaviors (48.19%)
	*M* (SE)	*M* (SE)	*M* (SE)
*Sexual behaviors*			
Deep kissing/making out	1.13 (.74)	2.71 (.32)	5.19 (.16)
Stimulation of genitals with hands	1.39 (.89)	2.04 (.47)	4.93 (.16)
Breast/nipple touching with hands	1.55 (.93)	3.34 (.28)	5.44 (.20)
Breast/nipple touching orally	1.20 (.97)	1.84 (.46)	4.54 (.21)
Genital-genital contact	1.16 (.73)	1.53 (.47)	4.01 (.20)
Oral-genital contact	1.10 (.63)	1.29 (.46)	4.24 (.16)
Oral-anal contact	0.25 (.23)	0.17 (.13)	0.88 (.15)
Using a sex toy with a partner	0.45 (.57)	0.79 (.27)	2.40 (.20)
Self-masturbation in the company of partner	0.52 (.34)	0.65 (.22)	2.33 (.19)
Penetration (with hands/fingers)	0.78 (.78)	1.34 (.44)	3.76 (.17)
Penetration (with penis, sex toy, or strap-on)	1.01 (.82)	1.42 (.49)	3.89 (.19)
*Affectionate behaviors*			
Hugging	4.90 (.24)	6.50 (.30)	6.86 (.04)
Holding hands	3.15 (.33)	5.60 (.60)	6.41 (.13)
Cuddling	3.38 (.45)	5.96 (.39)	6.71 (.09)
Spooning	2.01 (.85)	4.82 (.27)	6.03 (.17)
Massaging shoulders/back	1.76 (.34)	2.67 (.19)	4.07 (.18)
Foot massage	0.25 (.14)	0.76 (.13)	1.24 (.15)
Caressing face	2.18 (.72)	5.22 (.37)	6.17 (.15)
Kissing on the lips	4.12 (.72)	6.35 (.18)	6.85 (.05)
Kissing on the face	3.18 (.75)	6.31 (.35)	6.74 (.08)
Kissing on the head	2.43 (.62)	5.71 (.55)	6.30 (.18)
Sitting in lap	0.99 (.20)	2.10 (.36)	3.36 (.22)
Arm around partner	3.20 (.68)	5.78 (.33)	6.26 (.14)
Staring into eyes	2.45 (.96)	5.06 (.24)	5.85 (.15)

The scale for the PBI is as follows: 0 = Not at all, 1 = Once a month, 2 = Once every two weeks, 3 = Once a week, 4 = Twice a week, 5 = 3 to 4 times a week, 6 = Once a day, 7 = More than once a day

#### Sexual Satisfaction

Participants completed the Global Measure of Sexual Satisfaction (GMSEX; Lawrance & Byers, 1995), a 5-item measure. Each item uses a distinct bipolar 7-point scale (e.g., *good—bad, pleasant—unpleasant)*. We computed sexual satisfaction scores by summing all response options, with possible scores ranging from 5 to 35; higher scores indicate better sexual satisfaction (α = 0.93).

#### Relationship Satisfaction

Participants completed the short form of the Couple Satisfaction Index (CSI-4; Funk & Rogge, 2007), which consists of four items. The first item used a 7-point Likert scale ranging from 0 = *Extremely unhappy* to 6 = *Perfect*. All remaining items used a 6-point Likert scale ranging from 0 = *Not at all true* to 5 = *Completely true.* We calculated relationship satisfaction scores by summing all response options with possible scores ranging from 0 to 21; higher scores indicate better relationship satisfaction (α = 0.91).

### Data Analysis Plan

To address our aims and hypotheses, we conducted a LPA in Mplus (ver. 8.4) on the 24 sexual and affectionate behaviors. To include sexual and relationship satisfaction as distal outcomes in the LPA, we used the three-step Bolck-Croon-Hagenaars (BCH) approach for including distal outcomes (Vermunt, 2010). For Step 1, we examined the frequencies of all sexual and affectionate behaviors to look for potential issues. Because all behaviors were endorsed by at least 5% and no more than 95% of participants, we retained all 24 behaviors for the LPA. Then, we conducted a series of exploratory LPAs using the 24 final indicators (i.e., both sexual and affectionate behaviors). Based on current recommendations (Bauer, 2022; Lanza et al., 2013), we used several goodness-of-fit indices, tests of statistical significance, and profile distributions to determine the number of latent profiles based on the mean patterns of the items from the PBI. Specifically, we examined the Akaike Information Criterion (AIC; Akaike, 1973), the Bayesian Information Criterion (BIC; Schwarz, 1978), and entropy for goodness-of-fit, and the Lo-Mendell-Rubin Adjusted Likelihood Ratio Test (LMRT; Lo et al., 2001) and the Bootstrapped Likelihood Ratio Test (BLRT; Bauer, 2022) to test statistical significance. Within LPA, the LMRT and BLRT compare solutions to determine whether a *k* profile solution fits the data better than a *k*-1 profile solution. For instance, the LMRT and BLRT for the 3-profile solution test whether the 3-profile solution fits the data better than the 2-profile solution, with a statistically significant value suggesting a better fitting model. We also examined class sizes, distributions, and means of each physical behavior to determine the interpretability of each model. We also explored frequencies of the number of participants sorted into each profile to assist with interpretability. After exploring the profiles, models, and parameter constraints, we decided on our final model that provided the best fit for the latent profiles. In Step 2, we added the three dichotomous couple-gender configuration variables to our final model as auxiliary variables to test whether the proportions of these couple-gender configurations within each profile differed in comparison to the reference group configuration (i.e., man/woman). Finally, for Step 3, to assess associations between latent profiles and sexual and relationship satisfaction (i.e., examining profile means for satisfaction and comparing means across profiles), we used our final model (3-profiles with couple gender configuration) and included sexual and relationship satisfaction as two distal outcomes and used the BCH analysis to test whether sexual and relationship satisfaction means significantly differed by latent profile group (i.e., used the latent profiles to predict average satisfaction scores). In contrast to couple gender configuration, our analyses for sexual and relationship satisfaction compared all profiles’ satisfaction averages to one another, and thus do not have a reference group.

## Results

We report fit statistics for 2–5 profile solutions in Table 3. It is important to note that for smaller sample sizes, it becomes increasingly difficult to find stable solutions with many classes. Based on our results, the LMRT and BLRT suggested that a 2-profile solution fit better than a 1-profile solution (*p* < 0.001; *p* < 0.001). For the 3-profile solution the BLRT indicated that a 3-profile solution fit better than a 2-profile solution (*p* < 0.001), although the LMRT did not (*p* = 0.17). Similarly, for the 4-profile solution the BLRT indicated that a 4-profile solution fit better than a 3-profile solution (*p* < 0.001), although the LMRT did not (*p* = 0.71). Due to the small sample size, the BLRT and LMRT for the 5-profile solution was unreliable, as indicated by several error messages in the model output. Although the LMRT and BLMRT provided mixed results across profiles, the AIC and BIC continued to decrease (i.e., fit better) from the 2 through 4-profile solutions (see Table 3), which suggested a better fit for profile solutions 3 and 4. Entropy across the 2 through 5-profile solutions remained relatively stable and high (i.e., > 0.80; Bauer, 2022). Thus, we further used class distributions and considered profile interpretability to determine our final profile solution. The results suggested several interpretability errors based on estimated vs. observed profiles for the 5-profiles solution, so we dropped this solution as a best-fitting model. Compared to the 3-profile solution, the 4-profile solution had fewer participants in two of the four profiles, which hinders reliability and interpretation (two profiles were < 15% of the overall sample). Additionally, the mean frequencies of physical behaviors did not meaningfully differ for the 4-profile solution compared to the 3-profile solution. Thus, based on parametrics of fit, interpretability, and parsimony, we settled on a 3-profile solution model. Then, following a 3-step approach, we included the three couple gender configuration indicators as axillary variables in our model and subsequently entered sexual and relationship satisfaction as distal outcome variables for a BCH analysis (Asparouhov & Muthén, 2014). Of note, due to missing couple gender configuration data, we excluded four people from the final model.

**Table 3:** Fit statistics for 2–5 profile LPA solutions of physical behaviors

Fit Statistics	Number of Profiles			
	2	3	4	5
AIC	**31,072.75**	**29,875.90**	**29,832.28**	29,526.94
BIC	**31,119.84**	**29,941.74**	**29,911.62**	29,622.40
LMRT	2205.34***	916.19	406.77	352.91
BLRT	− 16,573.63***	− 15,294.86***	− 14,997.92***	− 14,793.14
Entropy	0.96	0.94	0.96	0.94

AIC: Akaike information criterion; BIC: Sample-size adjusted bayesian information criterion; LMRT: Lo-mendell-rubin adjusted LRT test; BLRT: Bootstrapped likelihood ratio test. Bolded results for the AIC and BIC indicate better-fitting model results, excluding the 5-profile results (as they were uninterpretable). For the LMRT and BLRT, ***p < .001

**Fig. 1:**
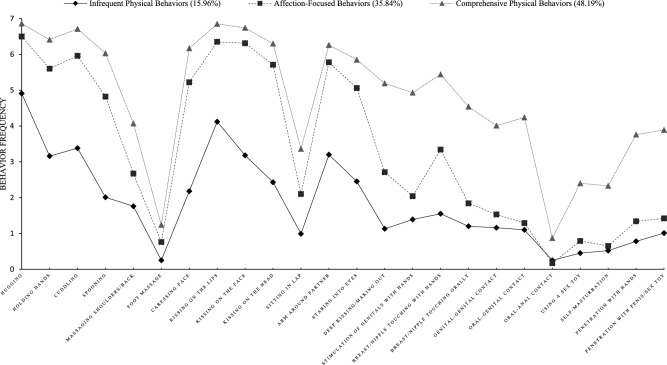
Specific behavior frequency means based on individual latent profiles scores on the y-axis reflect mean scores; 0 = Not at all, 1 = Once a month, 2 = Once every two weeks, 3 = Once a week, 4 = Twice a week, 5 = 3 to 4 times a week, 6 = Once a day, 7 = More than once a day

Figure 1 depicts the mean patterns of PBI items across the three latent profiles. Profile 1 comprised 15.96% of the sample (*n* = 53), and we labeled this profile Infrequent Physical Behaviors because people in this profile did not frequently engage in any affectionate or sexual behaviors, with the exception of moderate frequency of kissing and hugging. Profile 2 comprised 35.84% of the sample (*n* = 119), and we labeled this profile Affection-focused Behaviors because people in this profile engaged in frequent affectionate behaviors, but did not frequently engage in any sexual behaviors. Lastly, Profile 3 comprised 48.19% of the sample (*n* = 160), which we labeled Comprehensive Physical Behaviors, because people in this profile frequently engaged in almost every sexual and affectionate behavior.

### Couple Gender Configuration

We included couple gender configuration in our final model, using the Infrequent Physical Behaviors profile as the reference due to the low frequency of almost all physical behaviors in this profile. Results indicated no differences in profile membership by couple gender configuration (*p’s* > 0.05).

### Sexual and Relationship Satisfaction Outcomes

**Fig. 2:**
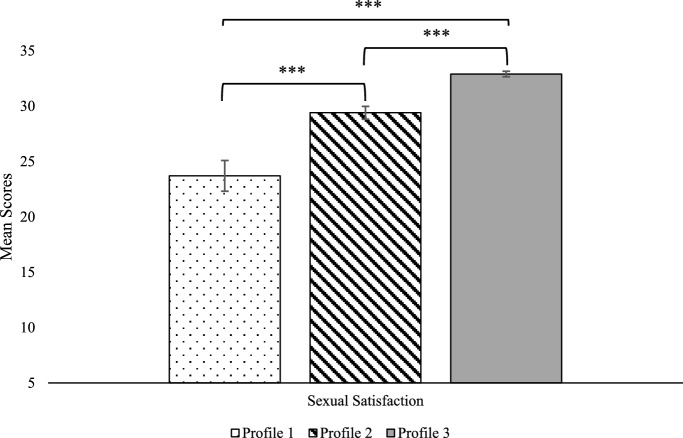
Sexual satisfaction mean comparisons across latent profiles. Possible scores for sexual satisfaction range from 5 to 35. ***p < .001

**Fig. 3:**
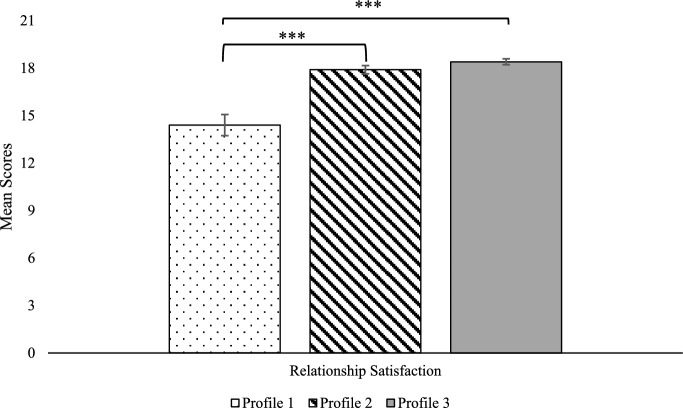
Relationship satisfaction mean comparisons across latent profiles. Possible scores for relationship satisfaction range from 0 to 21. ***p < .001

Sexual satisfaction and relationship satisfaction differed across all three profiles (see Table 4 and Figs. 2, 3). Participants in the Infrequent Physical Behaviors profile were less sexually and relationally satisfied than participants in the two other profiles. In addition, participants in the Comprehensive Physical Behaviors profile were more sexually satisfied than participants in the Affection-focused Behaviors profile, but did not differ on relationship satisfaction.

**Table 4:** Analyses comparing sexual and relationship satisfaction across latent profiles

	Sexual satisfaction			Relationship satisfaction		
	Profile 1 Infrequent physical behaviors	Profile 2 Affection-focused behaviors	Profile 3 Comprehensive physical behaviors	Profile 1 Infrequent physical behaviors	Profile 2 Affection-focused behaviors	Profile 3 Comprehensive physical behaviors
M (SE)	23.71 (1.39)	29.40 (.58)	32.93 (.25)	14.40 (.67)	17.91 (.26)	18.38 (.19)
vs. Profile 2	13.96***	–		23.29***	–	
vs. Profile 3	42.83***	30.19***	–	32.95***	2.17	

M: mean; SE: standard error. ***p < .001

## Discussion

Using a person-centered approach grounded in social exchange theory, we conducted a LPA to explore adults’ patterns of sexual and affectionate behaviors in romantic partner relationships with a sexual- and gender-diverse sample. Our results indicated three profiles: Infrequent Physical Behaviors, Affection-focused Behaviors, and Comprehensive Physical Behaviors. Further, profiles did not significantly differ as to whether participants belonged to a mixed-gender couple compared to any other couple-gender configuration. However, sexual and relationship satisfaction did differ by profile.

Prior research indicates that adults engage more frequently in certain sexual and affectionate behaviors than others (e.g., kissing, oral sex; Blumenstock et al., 2020), but we know of little research that explores a large number of physical behaviors—especially behaviors inclusive to LGBTQ+ individuals—and attempts to understand patterns of these behaviors. In particular, there is little research that examines affectionate behavior patterns, or whether these patterns are associated with relationship outcomes. Using LPA, we found three unique profiles of adults’ sexual and affectionate behaviors categorized by less frequent to more frequent physical behaviors. Although the Affection-focused Behaviors and Comprehensive Physical Behaviors profiles looked quite similar to each other in patterns of affectionate behavior, only the Comprehensive Physical Behaviors profile included frequent engagement in genital behaviors, whether by stimulation with hands, mouth, or penetration. Therefore, our results highlight distinct patterns of adults’ physical behaviors in long-term romantic relationships that may contradict heteronormative standards associated with typical physical behavioral progression (e.g., that physical behavior will progress from affectionate to increasingly sexual behaviors in a romantic relationship; Sassler & Michelmore, 2016). That is, many adults in our sample engaged in affectionate behaviors much more frequently than sexual behaviors, despite being in a long-term relationship.

### Adults in Mixed Gender Couples Had Similar Profiles to Adults in Other Couple Gender Configurations

Prior research suggests that sexual and affectionate behaviors differ by gender or by couple gender configuration. For instance, compared to women, men, particularly when in a relationship with another man, engage in a wider variety of sexual behaviors and engage in these behaviors more frequently (Diamond & Blair, 2018; Floyd, 2019). However, in the current study, adults in mixed gender couples did not differ in profile membership from adults in other couple gender configurations. This lack of difference suggests that adults—regardless of their gender presentation or socialization—frequently engage in a wide variety of physical behaviors, potentially as a way to sustain rewarding relationship exchanges given known benefits of physical behaviors to relationships (Byers & Wang, 2004; Sprecher et al., 2018). It is also possible that in our primarily LGBTQ+ sample, couple gender configuration may not have differentiated profiles as much as it might have in a primarily heterosexual, cisgender sample—the focus of most prior research on this topic. That is, having an LGBTQ+ identity may lead to more reflection and communication about their own sexuality and relationship with a romantic partner. However, it is also important to note that our four couple gender configuration groups do not fully represent the wide diversity of gender, and therefore the potential diversity of couple gender configurations that exists. For example, we did not differentiate cisgender and transgender participants, and it is possible that couples with one or two transgender individuals may engage in different patterns of behaviors than couples with only cisgender individuals (e.g., Dyar et al., 2020). Thus, future research should attempt to replicate current results with larger gender-diverse samples. In addition, to be as inclusive as possible we did not differentiate vaginal penetration from anal penetration, and these specific behaviors likely differ by sex-assigned at birth, and possibly by gender identity as well. Future research should consider how physical behavior profiles might differ by sex assigned at birth, and whether sex assigned at birth interacts with gender identity.

### How Sexual and Relationship Satisfaction Differ by Physical Behavior Profiles

Both social exchange theory and prior research with primarily heterosexual or women-only samples suggest that engaging in specific sexual (e.g., oral sex, penile-vaginal sex) and affectionate (e.g., hugging, kissing) behaviors is rewarding and associated with adults’ sexual satisfaction in their romantic relationships (Blumenstock et al., 2020; Byers & Wang, 2004; Frederick et al., 2017; Rausch & Rettenberger, 2021). Our results extend prior research and the generalizability of social exchange theory by including a gender-diverse sample and person-centered analyses, indicating that sexual and relationship satisfaction differ by patterns of adults’ sexual and affectionate behaviors. For instance, adults who frequently engaged in many sexual behaviors were more sexually satisfied than adults in the two profiles characterized by less frequent sexual behaviors. In contrast, adults in the profile that engaged in the least frequent affectionate behaviors were less relationally satisfied than adults in the two other profiles. These results support and inform social exchange theory by highlighting the rewarding nature of frequent engagement in many sexual and affectionate behaviors for maintaining adults’ sexual satisfaction, above and beyond behaviors examined in prior research (Blumenstock et al., 2020; Frederick et al., 2017; Rausch & Rettenberger, 2021). The fact that the affection focused and comprehensive behavior profiles were similar to each other on affectionate behaviors but differed on sexual behaviors, and did not significantly differ on relationship satisfaction, highlights that frequent and varied affectionate behaviors may be uniquely important for sustaining adults’ relationship satisfaction. For example, affection is often described as a primary building-block for initiating and sustaining most romantic relationships (Gulledge et al., 2007), which may be reflected by the lack of differences in relationship satisfaction between the Affection-focused and Comprehensive Physical Behaviors profiles.

Our findings also hold implications for clinicians working with adults in romantic relationships. Couples and individuals in romantic relationships commonly seek therapy or counseling for issues related to sexual and relationship satisfaction (Blair & Pukall, 2014; Metz & McCarty, 2007), but prior research often emphasizes addressing issues with adults’ sexual or affectionate behaviors separately or focuses only on a few specific behaviors (e.g., kissing, cuddling, oral sex; Blumenstock et al., 2020). Our findings suggest that clinicians should consider addressing adults’ sexual and affectionate behaviors together, and highlight the importance of a variety of affectionate behaviors in particular. For instance, clinicians working specifically on relationship satisfaction issues may want to focus on adults’ affectionate behaviors. Adults in either profile with less frequent sexual behaviors were more relationally satisfied than adults who engaged in a small range of affectionate behaviors. Because affectionate behaviors are often a staple of romantic partner relationships (Gulledge et al., 2007), encouraging adults to engage in many affectionate behaviors based on their personal preferences may uniquely benefit their relationship satisfaction. Thus, our results suggest that whether clinicians focus on addressing sexual and affectionate behaviors together or separately might depend on whether the couple’s issues concern sexual or relationship satisfaction, or both.

### Limitations

There are several limitations to the current paper. First, we solely explored adults’ physical behaviors with their longest or primary partner. It is possible that adults with multiple relationship partners might engage in more frequent physical behaviors than adults with only one partner. Therefore, our focus on the primary partner might not accurately capture their typical physical behaviors. Future research might consider adults’ specific behavior patterns across partners to understand whether behavioral patterns and feeling sexually or relationally satisfied differs for an individual across partners. In addition, we included only one partner per couple, but future research should consider collecting data from both partners to better understand the complex associations between both partners’ reports of physical behaviors and each partner’s satisfaction.

Second, although our sample size was robust enough to conduct LPA, due to the complex nature of this type of analysis and the number of latent profile indicators, our results must be interpreted with caution. Prior research indicates researchers should have a minimum of 200 participants to conduct LCA/LPA (Kyriazos, 2018). However, to prevent model error and small cell sizes, larger sample sizes are preferable (i.e., more than 300–400) when conducting complex analyses (e.g., analyses with more indicators or with distal outcomes). Thus, researchers could consider whether they can replicate these results with larger sample sizes. In addition, the association between profile membership and satisfaction may differ by couple gender configuration, but our sample size did not allow us to test for these differences. Future research with larger samples should consider such analyses to better identify how profiles of physical behaviors and couple gender configuration may interact in their association with satisfaction.

Third, the nature of identifying patterns in LPA means that associations between satisfaction and profiles include a range of behaviors. That is, associations with satisfaction indicate associations with a particular pattern, but cannot determine whether a specific behavior within that pattern drives the association.

Fourth, because the data are cross-sectional, we cannot determine directionality between adults’ patterns of physical behaviors and sexual and relationship satisfaction, and these are likely bidirectional. Certain patterns of physical behaviors likely lead adults to feel more sexually or relationally satisfied, but adults who are less sexually or relationally satisfied likely engage in certain patterns of physical behaviors due to their lack of sexual and/or relationship satisfaction. Therefore, clinicians might focus not only on evaluating satisfaction, but also may want to consider adults’ current physical behavior patterns as a potential barometer of current satisfaction. Future research should use longitudinal data to examine the temporal ordering of adults’ patterns of physical behaviors and their sexual and relationship satisfaction.

Lastly, the current sample is primary White, and our results may not generalize to adults with other racial/ethnic identities. Prior research indicates that experiences in romantic partner relationships often differ by race/ethnicity (e.g., Tornello, 2021), and it is possible that differences in sexual and relationship satisfaction by physical behavior profiles might vary by racial/ethnic identity. For example, adults with marginalized racial/ethnic identities may experience identity-related stigma and stress within their relationship, which may be associated with membership in profiles with less frequent physical behaviors (Tornello, 2021). Therefore, future research should include adults with other racial/ethnic identities to better understand how their sexual and relationship satisfaction differ by their physical behavior patterns. In addition, we did not collect data on recruitment method for each participant, so we cannot determine whether participants differed by recruitment source, thus limiting the generalizability.

### Conclusions

Our findings expand on prior research by examining specific physical behavior patterns in a primarily LGBTQ+ sample of adults in romantic partner relationships. Findings support social exchange theory, in that adults in profiles characterized by frequent affectionate behaviors were more sexually and relationally satisfied than adults in profiles characterized by less frequent affectionate behaviors, and adults in profiles characterized by frequent sexual behaviors were more sexually (but not relationally) satisfied than adults in profiles characterized by frequent affectionate but not sexual behaviors. Overall, results suggest the importance of examining adults’ patterns of physical behaviors, and highlight how clinicians might better approach adults’ challenges with physical behaviors and promote sexual and relationship satisfaction.

## Data Availability

Available upon request.

## Code Availability

Available upon request.
